# Exciton Coupling and Conformational Changes Impacting the Excited State Properties of Metal Organic Frameworks

**DOI:** 10.3390/molecules25184230

**Published:** 2020-09-15

**Authors:** Andreas Windischbacher, Luca Steiner, Ritesh Haldar, Christof Wöll, Egbert Zojer, Anne-Marie Kelterer

**Affiliations:** 1 Institute of Physical and Theoretical Chemistry, Graz University of Technology, NAWI Graz, Stremayrgasse 9, 8010 Graz, Austria; andreas.windischbacher@uni-graz.at (A.W.); luca.steiner@student.tugraz.at (L.S.); 2 Institute of Solid State Physics, Graz University of Technology, NAWI Graz, Petersgasse 16, 8010 Graz, Austria; 3 Institute of Physics, University of Graz, Universitätsplatz 5, 8010 Graz, Austria; 4 Institute of Functional Interfaces (IFG), Karlsruhe Institute of Technology (KIT), Hermann-von-Helmholtz Platz-1, 76344 Eggenstein-Leopoldshafen, Germany; ritesh.haldar@kit.edu (R.H.); christof.woell@kit.edu (C.W.)

**Keywords:** metal organic frameworks, SURMOF, absorption, emission, time-dependent density functional theory, aggregation

## Abstract

In recent years, the photophysical properties of crystalline metal-organic frameworks (MOFs) have become increasingly relevant for their potential application in light-emitting devices, photovoltaics, nonlinear optics and sensing. The availability of high-quality experimental data for such systems makes them ideally suited for a validation of quantum mechanical simulations, aiming at an in-depth atomistic understanding of photophysical phenomena. Here we present a computational DFT study of the absorption and emission characteristics of a Zn-based surface-anchored metal-organic framework (Zn-SURMOF-2) containing anthracenedibenzoic acid (ADB) as linker. Combining band-structure and cluster-based simulations on ADB chromophores in various conformations and aggregation states, we are able to provide a detailed explanation of the experimentally observed photophysical properties of Zn-ADB SURMOF-2: The unexpected (weak) red-shift of the absorption maxima upon incorporating ADB chromophores into SURMOF-2 can be explained by a combination of excitonic coupling effects with conformational changes of the chromophores already in their ground state. As far as the unusually large red-shift of the emission of Zn-ADB SURMOF-2 is concerned, based on our simulations, we attribute it to a modification of the exciton coupling compared to conventional H-aggregates, which results from a relative slip of the centers of neighboring chromophores upon incorporation in Zn-ADB SURMOF-2.

## 1. Introduction

Over the past two decades metal-organic frameworks (MOFs) have attracted significant attention due to their structural versatility and multitude of potential applications. They consist of metal/metal-oxo nodes connected by organic linker molecules and form highly regular, crystalline, and porous networks [1]. The huge variety of conceivable nodes and linkers has led to the synthesis of tens of thousands of different systems with specifically tuned properties [2]. Besides their established applications in areas like catalysis [3,4,5] gas storage [6,7,8] and gas separation [9,10] in recent years also their electronic and optical properties have attracted considerable interest [11,12,13,14,15]. Chromophores incorporated into MOFs have already been explored in sensing [16,17] and artificial light harvesting [18,19,20]. From a more fundamental point of view, an appealing aspect of MOFs in the context of optical spectroscopy is that they allow assembling chromophores at particularly well controlled relative positions [21,22] for example opening up the possibility of indirect bandgap formation, which is otherwise very uncommon in organic solids [23]. Furthermore, close stacking of chromophores in combination with long range order has long been a major target to control photon absorption [24,25], exciton diffusion [26,27,28,29], or luminescence properties [30,31,32,33,34].

Of crucial relevance in that context is the interaction of the individual chromophores with their surroundings, in particular with other chromophore molecules [35]. The stacking architecture of the molecules affects the energies of the excited states, the oscillator strengths of the transitions to these states and, consequently, the optical spectra of the assemblies. Here, the inclusion of chromophores into MOFs and related materials with their well-defined relative arrangement of organic linkers offers a particularly intriguing approach to tune their packing, allowing a targeted implementation of aggregation effects [22,36]. In this way, it is possible to realize chromophore conformations that significantly differ from the crystal structures of the neat bulk materials.

An advantageous side effect of the crystallinity of MOFs and their geometrically defined linker-node positions is their suitability for a detailed theoretical analysis [37]. Their high structural quality and the possibility to produce highly oriented and crystalline thin films (which is often not easy to achieve for neat organic chromophores), makes them particularly well suited for validating theoretical approaches, which can then be used for designing new materials on the computer. This can significantly reduce the necessary experimental efforts and aids the development of high-performance materials [38,39,40]. In fact, an in-depth understanding of the properties of excited states gained in such simulations is of direct relevance for processes like exciton migration, exciton separation, or exciton recombination. These processes crucially impact the applicability of the studied materials in devices like solar cells, photodetectors or light emitting diodes. Nevertheless, relatively little attention has been paid to understanding optically excited states in MOFs from a theoretical perspective, which we intend to change in the present study.

As far as the specific chromophore is concerned, we, here, focus on 4,4′-(anthracene-9,10-diyl)dibenzoic acid (ADB). The carboxylic acid groups allow the connection to metal nodes as a prerequisite for the formation of a MOF, while the core of the molecule consists of the widely used fluorescence standard 9,10-diphenylanthracene (DPA) [41,42], which serves as a prototypical example for a π-conjugated, acene-based dye [43,44,45,46]. The first excited state of DPA is known to have a long lifetime and high quantum yield, making ADB-linked systems an interesting choice for studies of the optical properties of MOFs [47,48].

**Figure 1:**
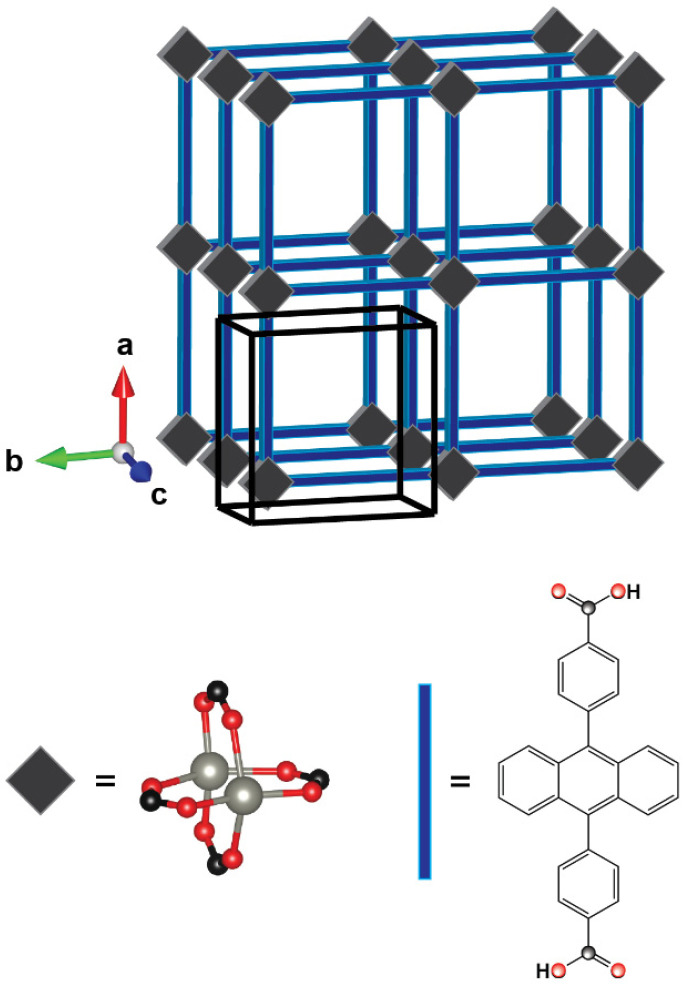
Schematic structure of Zn-ADB SURMOF-2; grey squares represent the Zn-paddlewheel, which is bridged by four carboxylic acids of the ligand (see bottom left panel, Zn ions grey); blue rods represent the anthracene-dibenzoic acid linker, where the acidic H is shown for completeness; the carboxylic linkage groups in the paddle wheel and in the molecular skeletal formula are indicated with the same color code: black: carbon, red: oxygen; the tetragonal unit cell is marked with black lines, the unit cell axes are labelled as used in the text.

The chosen model system, Zn-SURMOF-2, consists of Zn^2+^ ions forming paddlewheel type secondary building units, which are linked by ADB. When growing the framework with layer-by-layer liquid-phase-epitaxy on a functionalized substrate, it builds two-dimensional square grid networks, which are stacked in an AA fashion (Figure 1) [49]. The unit cell length along the stacking axis, *c*, amounts to 5.82 Å, while the square grid axes (*a* and *b*) have a length of 19.81 Å. The anisotropy of the unit cell, for example, results in a pronounced anisotropy of excited-state energy transfer, which is efficient only along the stacking direction -*c*, [50,51]. As far as the optical properties of the ADB molecules incorporated into the MOF are concerned, it has been observed that the absorption spectrum of Zn-ADB SURMOF-2 is hardly shifted compared to that of the ADB chromophore in solution (absorption maximum at 3.32 eV in ethanol vs. 3.27 eV in the MOF [51]. Conversely, in the experiments the emission maximum shifts considerably from 2.87 eV in ethanol to 2.63 eV in the MOF, where the peak positions have been extracted from the experimental spectra contained in the Supplementary Material (SI.9). These correspond to the data contained also in [51]. In that paper, the red-shift in emission has been attributed to the formation of excimers.

In the following, we will use quantum-mechanical simulations to provide an in-depth analysis of the excited state properties of ADB both as isolated chromophore and when π-stacked in a well-defined manner inside a coordinatively linked MOF. In particular, we will address the questions, (i) why there is a weak red- shift in the absorption spectrum despite the primarily H-aggregate type exciton coupling in the MOF and (ii) what triggers the red-shift of 0.24 eV of the MOF emission compared to the emission of individual chromophore in solution, which increases the energy difference between absorption and emission maxima from 0.45 eV in solution to 0.64 eV in the MOF. To answer these questions, we will analyze (a) the impact of the solvent, (b) the role played by the packing-motif in the MOF (H- vs. J-aggregates and the consequence of the relative arrangement of the chromophores), (c) the impact of changes in the molecular conformation, and (d) the nature of the excited states dominating absorption vs. emission processes in the MOF. In the following discussion, we will focus on the purely electronic properties of the excited states, acknowledging that for explaining certain details of the absorption and emission spectra of molecular aggregates one also has to include electron-phonon coupling effects [52], as, for example, reviewed for aggregates of para-distyrylbenzene by Gierschner et al. [53].

## 2. Results and Discussion

### 2.1. Molecular Properties and Solvent Effects

**Figure 2:**
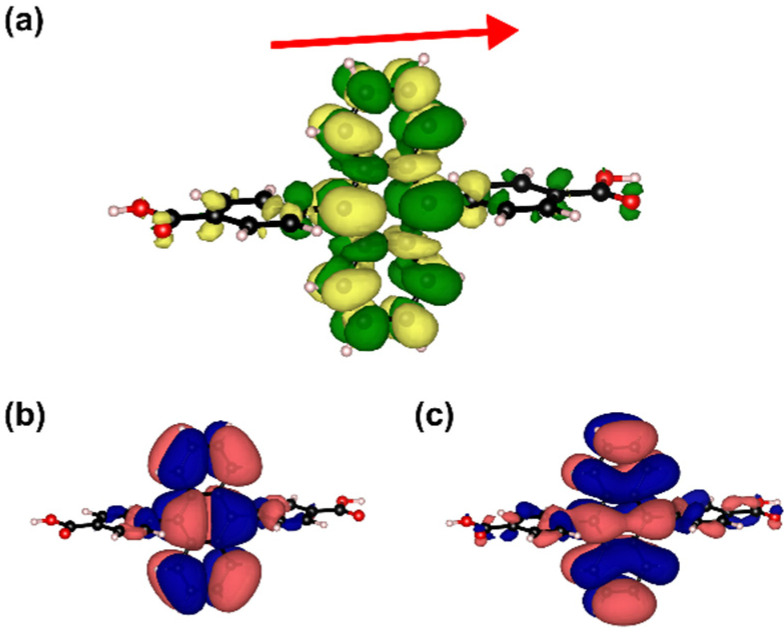
(**a**) Isodensity (isovalue 0.0005) plot showing the PBE0/def2-TZVP//def2-SVP calculated transition density for the S_0_→S_1_ excitation of the ADB molecule in its ground-state configuration. The red arrow denotes the direction of the corresponding transition dipole vector; (**b**) isodensity plots (isovalue 0.01) of the highest occupied molecular orbital (HOMO) and (**c**) lowest unoccupied molecular orbital (LUMO) of ADB.

As a first step, we analyze the situation for an isolated ADB molecule. The transition to the lowest excited singlet state, S_1_, is optically allowed and the state is primarily described by an excitation from the highest occupied molecular orbital, HOMO, to the lowest unoccupied molecular orbital, LUMO. Both orbitals are largely localized on the central anthracene unit. This is also observed for the transition density associated with the S_0_→S_1_ transition (see Figure 2). It can be explained by a nearly complete breaking of conjugation between the anthracene core and the attached phenylenes, which is a consequence of an almost perpendicular arrangement of the π-planes in the respective units (with a twist angle of 84°). Consequently, when comparing the lowest excitation energies in anthracene and in the isolated ADB molecule, one obtains an only rather small red-shift of 0.15 eV (see Table 1). The reason why there is any shift at all is a minor spread of the excited state onto the phenylenes, as can be inferred from the shapes of the frontier orbitals and the transition density (see Figure 2). Consistently, the electron withdrawing carboxylic acid substituents have an only very weak impact on the optical properties of ADB. This can be concluded from the data for diphenylanthracene (DPA) (see Table 1), which essentially coincide with those for ADB. Finally, it should be mentioned that the transition dipole for the lowest excited state in all molecules discussed in this paragraph is parallel to the short molecular axis of the anthracene moiety (cf. Figure 2).

The strong localization of the S_1_ state in the apolar center of the ADB molecule also explains, why including solvents of varying polarity in the calculations has hardly any impact on the lowest excited state (see Table 1). This theoretical finding is also consistent with experimental studies on DPA, which show that decreasing the polarity of the solvent from ε = 24 (ethanol) to ε = 2.4 (toluene) increases the excitation energy by at most 0.05 eV [55,56,57]. As a consequence, solvent effects are ignored for the present system and will not be accounted for in the following discussion.

As far as the emission properties of ADB are concerned, we calculate an energy of 2.78 eV for the purely electronic S_1_→S_0_ transition (calculated for the S_1_ equilibrium geometry). This corresponds to a rather large shift of ~ 0.55 eV between the absorption and emission maxima. The magnitude of the shift can be explained by a reduced twist angle of the phenylene rings relative to the anthracene in the excited state equilibrium geometry (56° in S_1_ vs. 84° in S_0_ geometry), which results in an increase of conjugation. Overall, comparing the results in Table 1 shows that for the isolated molecule the simulations agree exceptionally well with the experiments. Thus, in the following we will focus on understanding the situation, when the ADB linkers are incorporated into the MOF.

### 2.2. Formation of H- and J-Aggregates in Zn-ADB SURMOF-2: Anthracene Dimers as Model Systems

For understanding the excited states of ADB incorporated into the MOF, as a first step, it is useful to analyze possible exciton couplings between the chromophores in the MOF structure. Depending on the relative alignment of the transition dipoles on adjacent chromophores, H-type or J-type aggregates are formed in a solid-state assembly. In H-type aggregates, the transition dipoles on adjacent chromophores are aligned in parallel, whereas J-type aggregates are formed with a head-to-tail alignment of the transition dipoles. In both aggregate types, the first excited state is shifted to lower energies compared to the isolated chromophore, but only in J-type aggregates this excited state is optically allowed, resulting in a red-shift of the absorption spectrum. In H-type aggregates, the first optically allowed state is typically found at energies higher than the parent state in the isolated chromophore, which results in a blue-shift [58].

For the following, discussion of the fundamental aspects of exciton coupling in Zn-ADB SURMOF-2 we will replace the ADB chromophores by anthracene units to simplify the interpretation. This does in no way affect the key conclusions regarding exciton coupling that will be discussed in this section and is justified by the strong localization of the excited state on the anthracene moiety in the isolated ADB molecule (vide supra).

**Figure 3:**
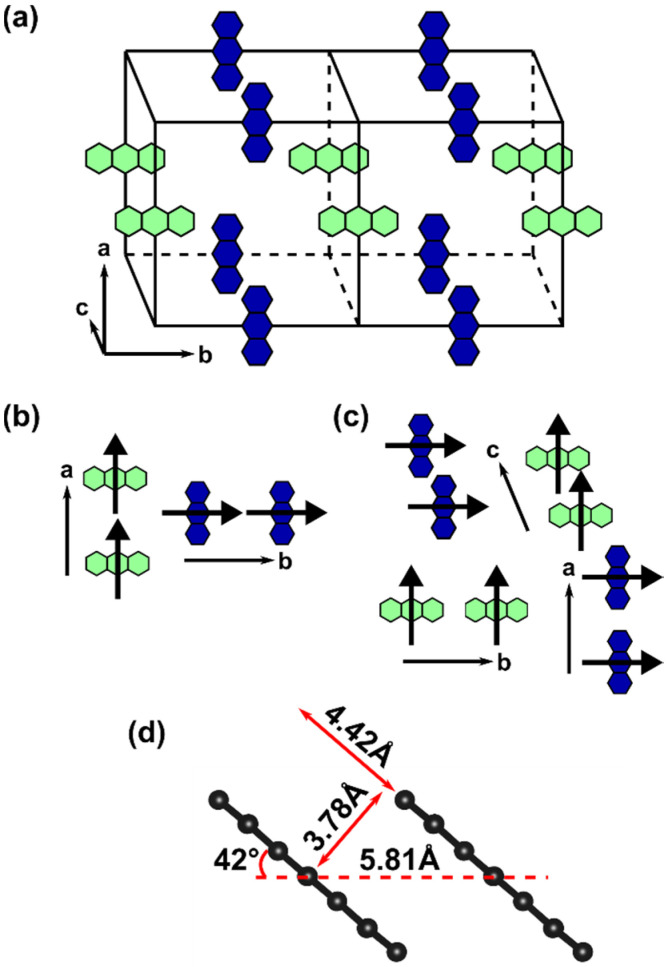
(**a**) Relative arrangement of anthracene units (blue and green) in Zn-ADB SURMOF-2; (**b**) anthracene units forming J-aggregates and (**c**) H-aggregates; the respective S_0_-S_1_ transition dipole moments of the individual chromophores are depicted as bold arrows; (**d**) relative alignment of neighboring anthracene units in the equilibrium structure of Zn-ADB SURMOF-2 arranged along the *c*-direction with a center-to-center distance of 5.81 Å. The distance between neighboring π-planes and the slip of the chromophores is also shown.

Both aggregate types discussed above can be found in Zn-ADB SURMOF-2, as schematically shown in Figure 3: In *a*-direction, one observes J-type (green molecules) as well as H-type aggregates (blue molecules). The center-to center distance between the chromophores in both cases amounts to 19.80 Å. This is so large that the effect of exciton coupling becomes vanishingly small, resulting in a negligible splitting between the lowest excited states (see Table 2). For symmetry reasons, exactly the same situation as in *a*-direction is also found in *b*-direction (where now neighboring blue molecules form J-aggregates and neighboring green molecules form H-aggregates). The situation is fundamentally different in *c*-direction, where all nearest-neighbor molecules form H-aggregates. As in this direction the molecules are rather close, the impact of the coupling becomes noticeable. Thus, in the following we will be exclusively concerned with H-aggregates stacked in *c*-direction. Notably, here the center to center distance of 5.81 Å in the equilibrium structure of the MOF corresponds to a distance of 3.78 Å between neighboring π-planes of the anthracenes, as illustrated in Figure 3d. This is a consequence of a tilting of the anthracenes, which also results in a slip of the centers of neighboring chromophores parallel to the π-planes by 4.42 Å.

For the *c*-stacked, H-aggregate-type dimer of anthracene, the splitting between the lowest excited state and the first state with appreciable oscillator strength amounts to 0.39 eV (see Table 2). Increasing the number of interacting chromophores somewhat increases the splitting (see last entry in Table 2), but considering the rather short distance between the neighboring π-planes, this splitting still appears rather small. Moreover, in a typical H-aggregate one would expect the oscillator strength for the excitation into the S_1_ state to be exactly zero for symmetry reasons.

**Figure 4:**
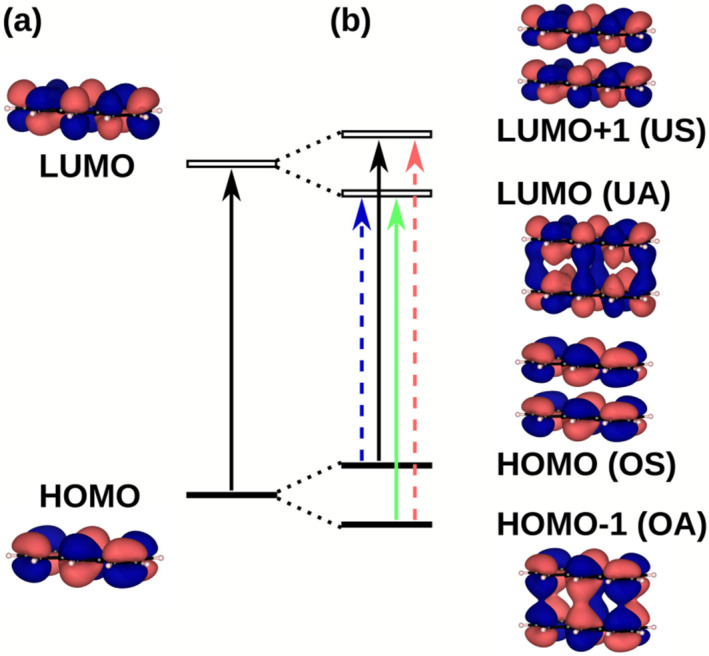
(**a**) HOMO and LUMO of anthracene, and (**b**) splitting of HOMO and LUMO of a cofacial anthracene dimer with the resulting excitations (allowed = solid arrow, forbidden = dashed arrow); symmetry labels of the orbitals in bracket result from the linear combination of the orbitals of the single molecule (S = symmetric linear combination, A = antisymmetric linear combination, O = occupied, U = unoccupied). The transitions are assigned as follows: allowed transitions S_b_ = OA→UA (green), and S_c_ = OS→US (black), forbidden transitions S_a_ = OS→UA (blue), S_d_ = OA→US (red).

To rationalize these findings, as a first step the excited state structure of a cofacial (i.e., not slipped) anthracene model dimer shall be discussed: In the dimer, hybrid orbitals are formed from the molecular HOMOs and LUMOs of each of the molecules. In a single-particle picture, four excitations between these orbitals are possible (see Figure 4). For symmetry reasons, two of these excitations are optically allowed and two of them are forbidden (solid vs. dashed arrows in Figure 4). In the actual time dependent density functional theory (TD-DFT) calculations the single-particle excitations mix. This yields four excited states, which can be characterized by linear combinations of either the allowed or the forbidden single-particle excitations. This is shown in the first entry of Table 3 (slip 0.0 Å) for the two cofacial anthracene molecules. In the following, the four excited states will be denoted as S_a_, S_b_, S_c_, and S_d_. Here, S_a_ refers to an excited state dominated by the positive linear combination of the forbidden single-particle transitions OS→UA and OA→US. In this context, O denotes to the highest occupied and U to the lowest unoccupied orbital of the dimer with a specific symmetry, where S and A specify, whether the dimer orbitals are symmetric (i.e., positive) or antisymmetric (i.e., negative) linear combinations of the orbitals of the individual molecules (see Figure 4). S_b_ refers to the positive linear combination of the allowed transitions OA→UA and OS→US and S_c_ and S_d_ denote the negative linear combinations of the respective transitions (see last column of Table 3). In line with the involved single-particle excitations, transitions to states S_a_ and S_d_ are strictly optically forbidden (see oscillator strengths in Table 3), while excitations to states S_b_ and S_c_ are, in principle, optically allowed, although in our simulations the oscillator strength for excitations into S_b_ are consistently much smaller than for excitations into S_c_. As a consequence, the position of S_c_ determines the position of the first peak in the absorption spectrum.

**Figure 5:**
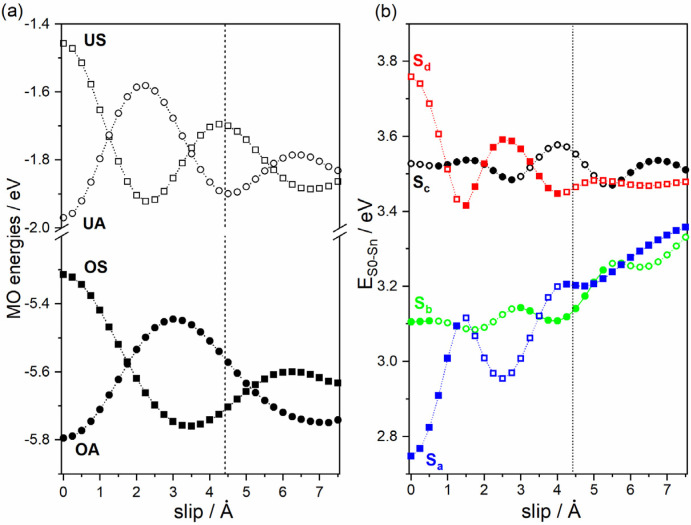
(**a**) Dependence of the energies of the two highest occupied and two lowest unoccupied orbitals, of anthracene dimers as a function of the slip between the centers of the molecules calculated of at the PBE0/def2-TZVP//def2-SVP. The orbitals are named employing the following nomenclature (according to Figure 4): S = symmetric linear combination, A = antisymmetric linear combination, O = occupied, U = unoccupied. (**b**) Analogous dependence of the energies of the two lowest symmetry-forbidden excitations (to states S_a_ and S_d_) and symmetry-allowed excitations (to states S_b_ and S_c_). S_a_, S_b_, S_c_ and S_d_ are described by superpositions of one-particle excitations according to Table 3. As detailed in the main text, S_a_ and S_d_ are dominated by the same single-particle excitations. The same applies to S_b_ and S_c_. Therefore, these pairs are plotted using the same symbols. Filled symbols specify a larger weight for the OS→UA (in the case of S_a_ and S_d_) and for the OA→UA single particle excitations (in the case of S_b_ and S_c_), while open symbols specify that the respectively other single particle excitation dominates (OS→UA for S_a_ and S_d_ and OS→US for S_b_ and S_c_). The vertical line indicates the slip of 4.42 Å that occurs in Zn-ADB SURMOF-2 (see Figure 3d).

For the cofacial anthracene dimer (first entry in Table 3), the expected situation for a conventional H-aggregate is recovered: The optically forbidden S_a_ state is lowest in energy and the first state with appreciable oscillator strength (S_c_) lies 0.78 eV above the first excited state. To understand the different properties of the anthracene dimer in the H-aggregate configuration adopted in Zn-ADB SURMOF-2 discussed before, one has to consider the slip of the centers of neighboring chromophores in the MOF (Figure 3d): As discussed by Kazmaier and Hoffmann for simple model systems and for perylene [59] (and later found for a variety of organic semiconductors [60,61,62]), displacing the centers of coplanar molecules relative to each other results in a periodic variation of the splitting of the respective hybrid orbitals as a function of the displacement. This occurs due to the symmetry of the individual orbitals (see Figure 5a). Moreover, because of the decrease of the spatial overlap of the molecules with increasing displacement, the amplitude of the oscillations decreases. These changes of the orbital energies cause also variations in the energies of the above-mentioned four excited states, as shown in Figure 5b. These variations do not directly coincide with the variations for the orbital energies, which has two reasons: First, due to the different nodal patterns of the HOMO and LUMO (compare Figure 4), the slips at which the splitting between the HOMO and the HOMO − 1 vanishes differs from the slips at which the same occurs for the LUMO and LUMO + 1. Second, the dominant single-particle excitation describing specific excited states changes with the displacement, as shown by the filling of the symbols in Figure 5 (for details see figure caption).

Notably, the slip-induced changes in excitation energies are large enough for the order of the states to change as a function of the displacement. In fact, for a slip of 4.42 Å (the value obtained in Zn-ADB SURMOF-2, indicated by a vertical line in Figure 5) the optically weakly allowed S_b_ state comes to lie lowest in energy instead of the strictly symmetry-forbidden S_a_ state. This is reminiscent of the situation in crystals of dicyanodistyrylbenzene based molecules, where static symmetry-breaking renders the lowest excited state of H-aggregate coupled chromophores optically allowed [63]. Moreover, the splitting between the lowest excited state and the first state with appreciable oscillator strength (S_c_) decreases by essentially a factor of two between the cofacial dimer (slip 0.0 Å) and the dimer in the Zn-ADB SURMOF-2 configuration (4.42 Å, see Table 3). This explains the somewhat unexpected excited state properties of the anthracene-dimer extracted from the Zn-ADB SURMOF-2 structure (vide supra). In passing we note that especially the change of the order of the states due to the slip will become relevant later, when discussing the emission properties of Zn-ADB SURMOF-2.

Still, there is one aspect of the calculations on the anthracene H-aggregates which is at variance with the experimental observation for Zn-ADB SURMOF-2: The simulations predict a blue shift of the absorption maximum (by 0.12 eV for the tetramer in Table 2 compared to the isolated molecule), while in the experiments a minor red shift of the absorption peak by 0.05 eV is observed (see Table 1). To understand that, one has to go beyond representing the chromophores in the simulations by anthracene units. In particular, one has to study to what degree the conformations of the actual ADB units change upon incorporation into Zn-SURMOF2.

### 2.3. Impact of the Chemical Linkages and The Solid-State Conformation on the Optical Properties of ADB in Zn-ADB SURMOF-2

**Figure 6:**
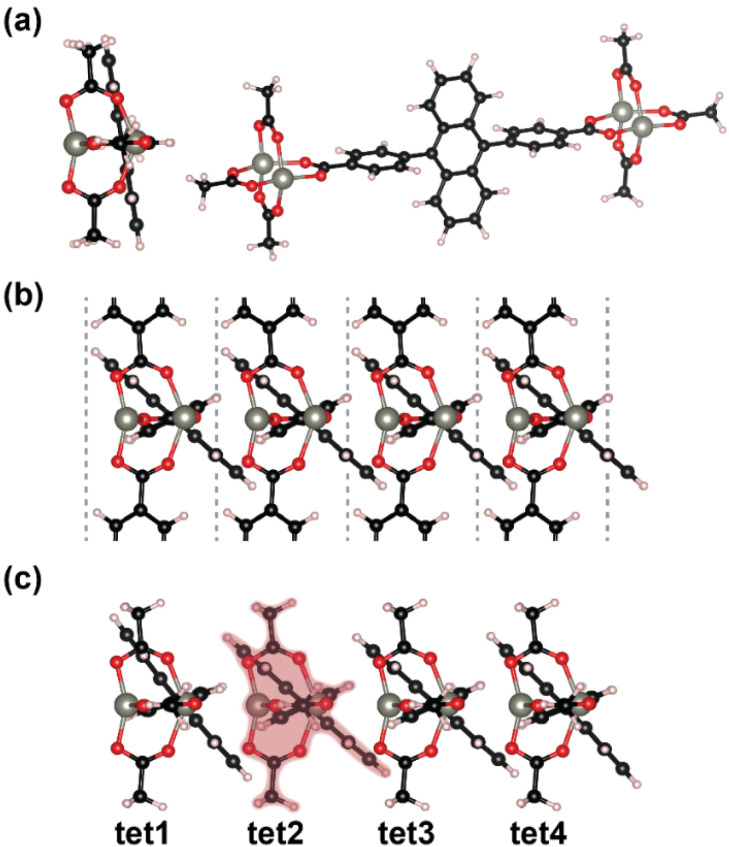
(**a**) Calculated ground state geometry of a single node-linker unit, ^opt^(pw-ADB-pw)_1_, (**b**) detail of the periodically calculated structure (the grey dashed lines indicate the unit cell boundaries), (**c**) optimized geometry of a tetramer, ^opt^(pw-ADB-pw)_4_; the central unit most similar to the periodic conformation (tet2) is highlighted in red. Grey: Zn ions, red: oxygen atoms, black: carbon atoms, white: hydrogen atoms.

As a first step towards answering that question, the impact of the bonding of the ADB chromophore to the metal nodes is assessed. For this purpose, we optimized a single ADB molecule suspended between two Zn-paddle wheels (pw) with the Zn and O atoms fixed to the positions they adopt in the periodic structure of the MOF discussed below. The Zn-nodes are additionally saturated by three acetate groups per paddlewheel (see Figure 6a). This structure in the following will be referred to as ^opt^(pw-ADB-pw)_1_, where the subscript denotes that only a single ADB unit is considered and the superscript refers to a full geometry optimization (where only the Zn and O atoms are fixed). This geometry optimization yields a structure very similar to the isolated ADB molecule with bond angles within 0.5 degrees and bond lengths within 0.02 Å (cf. Supplementary Material, SI.2). The changes in bond-lengths are primarily triggered by fixing the Zn-Zn distance between the paddle wheels. Also, the nature and energy of the lowest excited state are very similar to those in the isolated molecule (cf., Table 1 and Table 4). This supports the finding from above that terminal substituents have essentially no impact on the lowest excited states of the chromophores. It also implies that for the present combination of chromophore and metal node, there is no “through-bond” electronic coupling (like in certain electrically conductive MOFs [14]) between adjacent chromophores in *a*- and *b*-direction.

One of the crucial aspects not captured by the ^opt^(pw-ADB-pw)_1_ model system is, how the conformation of the chromophore is changed by the neighboring linkers in *c-* direction. To capture the influence of the neighbors, as a first step we optimized the structure of the 3D MOF employing periodic boundary conditions. For reasons explained in the Methods Section, this has been done using the PBE functional [64]. From the periodic structure we extracted a tetramer cluster repeated in *c*-direction (consisting of four ADB molecules bonded to two saturated Zn-paddle wheels). This cluster was then further optimized with the PBE0 functional [65] (like in the molecule-based simulations), fixing the positions of the Zn atoms. This is done to deal with geometries (in particular bond lengths) obtained at a level of theory consistent with the previously discussed simulations. The structure of that tetramer cluster, ^opt^(pw-ADB-pw)_4_, is shown in Figure 6c. In this cluster, the geometries of the outermost (pw-ADB-pw) units are impacted by edge effects, but comparing the structures shown in Figure 6b,c, one sees that the two central units adopt an arrangement fully consistent with the periodic geometry optimizations. A more quantitative analysis of the results shows that all twist angles agree to within less than 1° and that bond lengths are within 0.004 Å compared to the values from the periodic simulations (for more details see Supporting Information SI.4). Thus, we used one of these units (viz tet2 in Figure 6c) as the basic building block for the model systems used in the following. The structures constructed using this “cut-out” monomer will be denoted as ^cut^(pw-ADB-pw)_n_, where the index n denotes the number of repeating units.

The geometric changes of all “cut” structures compared to optimized monomer structure, ^opt^(pw-ADB-pw)_1_, arise from the impact of the neighboring ADB chromophores inside the MOF. These changes primarily concern the twist of the anthracene and phenylene moieties of ADB relative to the plane in which the neighboring Zn atoms are arranged (compare Tables S3 and S4 in the Supplementary Materials). In the optimized monomer, ^opt^(pw-ADB-pw)_1_, the phenylenes are essentially in the plane of the Zn atoms (see Figure 6a). Such a conformation is prevented in the 3D periodic structure by steric constraints, as can be inferred from the structure shown in Figure 6b. In fact, for phenylenes in the plane of the Zn atoms, the H atoms on neighboring rings would come much too close to each other. Consequently, the phenylenes are twisted by 24° relative to the Zn plane in the periodic conformation. Also, the orientation of the anthracene units is significantly modified. While the anthracene plane is nearly perpendicular to the Zn-plane in ^opt^(pw-ADB-pw)_1_ (at 82°), the twist between the two planes is reduced to 42° in the periodic structure. This reduced anthracene-Zn plane twist is primarily a result of van der Waals interactions between neighboring anthracenes trying to reduce the distance between the π-planes of the molecules (as shown in SI.3 contained in the Supplementary Materials). Most importantly, as a consequence of that also the angle between the phenylene and anthracene planes is reduced from 81° in ^opt^(pw-ADB-pw)_1_ to 66° in the periodic structure.

To study the impact of the changes in twist angles, we first discuss the properties of a single “cut” chromophore, ^cut^(pw-ADB-pw)_1_, (see highlight in Figure 6c). The energy of the lowest excited state of ^cut^(pw-ADB-pw)_1_ is distinctly red-shifted (by 0.17 eV) compared to the fully optimized monomer, ^opt^(pw-ADB-pw)_1_, and the oscillator strength is significantly increased (see Table 4). This can be attributed to the change of the conformation of the chromophore when incorporated into the MOF, where the main aspect is that the reduced twist between the phenylenes and the anthracene in the ADB unit results in an increased conjugation (see Supplementary Material SI.5).

### 2.4. The Final Absorption Spectrum of Zn-ADB SURMOF-2: Combining Conformational Changes and Aggregate Formation

**Figure 7:**
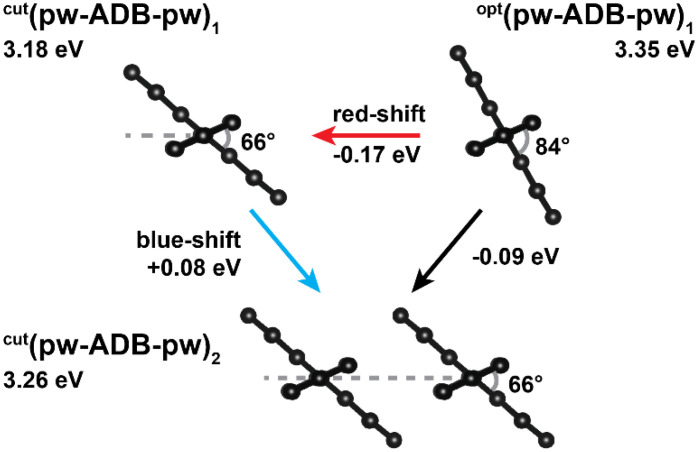
Schematic depiction of the interplay between aggregational and conformational effects on the optical properties of Zn-ADB SURMOF-2: the Zn-nodes are omitted for clarity and indicated by the grey dashed lines connecting two neighboring nodes.

In the previous sections we have shown that when assembling ADB chromophores into Zn-SURMOF-2, there are two competing effects regarding the change in the absorption spectrum: On the one hand, there is a red-shift of the lowest excited state in the MOF due to the conformational changes triggered by inter-linker interactions and a related increase of conjugation (see Section 2.3 and horizontal arrow in Figure 7). On the other hand, H-aggregate formation in *c*-direction causes a blue-shift of the first strongly allowed state, as discussed in Section 2.2 (see Table 2). This raises the question, how the combination plays out in the actual MOF, in which exciton coupling and conformational changes happen simultaneously. To address that, we constructed a dimer and a tetramer by assembling the “cut” monomer entities described above. This yielded ^cut^(pw-ADB-pw)_2_ and ^cut^(pw-ADB-pw)_4_, where the former system has the advantage that its properties in terms of the nature of orbitals and excited states can be discussed in analogy to the situation of the anthracene dimers from Section 2.2. Indeed, it turns out that the nature and order of the excited states in ^cut^(pw-ADB-pw)_2_ are equivalent to those of the anthracene dimer with a slip of 4.42 Å (cf., Table 3 and Table 4): In the TD-DFT simulations on ^cut^(pw-ADB-pw)_2_, the lowest excited state displays S_b_ character (the negative linear combination of OA→UA and OS→US transitions), while the state with the largest oscillator strength is the third excited state possessing S_c_ character (the respective positive linear combination). Compared to the slipped anthracene dimer, the oscillator strengths of both states are increased, which is due to the spreading of the transition density onto the phenylene units (cf., Supplementary Material SI.5; see also comparison of the properties of anthracene and ADB in Table 1). This effect is a consequence of the reduced twist between the anthracene and the phenylene unit, when the ADB chromophores are incorporated into the MOF. As the oscillator strength associated with the S_c_ state is by a factor of more than five higher than that of the S_b_ state, the energy of S_c_ determines the position of the first absorption peak. Due to the dimer formation, in ^cut^(pw-ADB-pw)_2_ this state is slightly blue-shifted by 0.08 eV compared to the corresponding monomer ^cut^(pw-ADB-pw)_1_ (see blue arrow Figure 7 and Table 4). Combining this blue shift by 0.08 eV with the red-shift by 0.17 eV between ^opt^(pw-ADB-pw)_1_ and ^cut^(pw-ADB-pw)_1_ due to conformational changes yields the overall red-shift of 0.09 eV between an isolated ADB molecule and ^cut^(pw-ADB-pw)_2_. This is schematically shown by the black arrow in Figure 7.

A similar situation is obtained when calculating the excited states of the tetramer, ^cut^(pw-ADB-pw)_4_. (see Table 4). Again, the lowest exited state is red-shifted compared to the monomer and states at higher energies dominate the absorption spectrum due to their larger oscillator strengths. The state with the highest oscillator strength amongst the first 20 excited states (S_14_) is even somewhat further blue-shifted than in ^cut^(pw-ADB-pw)_2_. Calculating a theoretical absorption spectrum from a superposition of Gaussian peaks with full widths at half maximum (FWHM) of 0.30 eV centered at the energies of the excited states of the tetramer and scaled by their oscillator strengths yields an absorption maximum at 3.29 eV (cf. Supplementary Material SI.7). This is only slightly higher than the experimental absorption maximum at 3.27 eV (see Table 1). In passing we note that an unambiguous signature of the weakly allowed S1 state in ^cut^(pw-ADB-pw)_2_ and ^cut^(pw-ADB-pw)_4_ cannot be identified in the experimental spectra, as discussed in more detail in the Supplementary Material (SI.9).

Overall, the above considerations explain, why the first absorption peak in Zn-ADB SURMOF-2 is not blue-shifted but rather red-shifted compared to the isolated ADB chromophore in solution in spite of the formation of H-aggregates. What remains to be explained is the significant shift of 0.64 eV between the absorption and emission maxima in Zn-ADB SURMOF-2.

### 2.5. Explaining the Red-Shifted Emission of ADB Molecules Incorporated into Zn-ADB SURMOF-2

**Figure 8:**
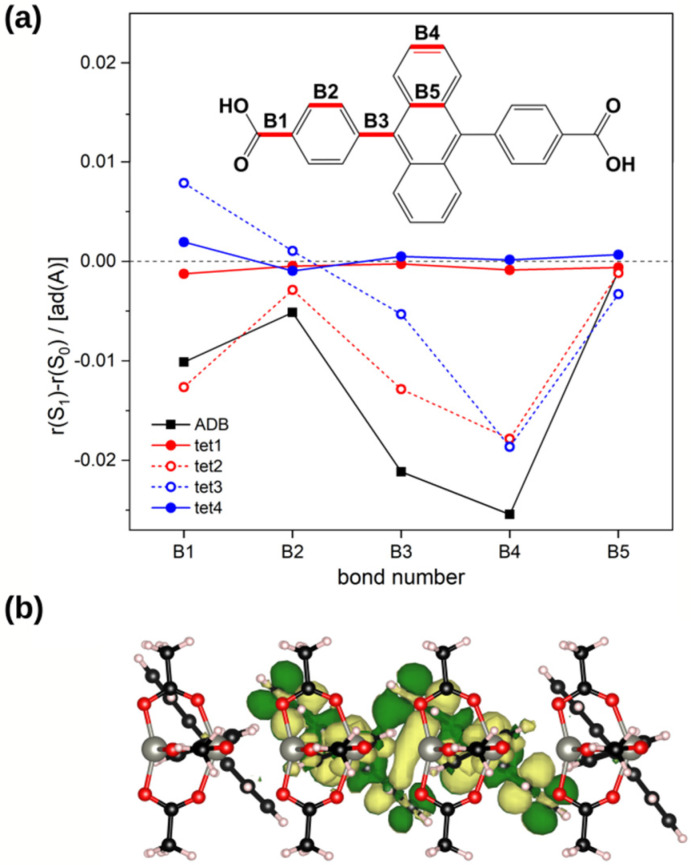
Changes in bond lengths between the ground- and excited state configurations and shape of the transition density associated with the first excited state calculated to determine the localization of that excited state [66,67]. Panel (**a**) shows the difference of the bond lengths of bonds B1-B5 between ground and first excited state for (pw-ADB-pw)_4_ and for an isolated ADB molecule. The labelling of the bond lengths is explained in the inset. All structures were optimized at the PBE0/def2-SVP+D3 level. Panel (**b**) contains the transition density associated with the first excited state of ^S1^(pw-ADB-pw)_4_ calculated with PBE0/def2-TZVP//def2-SVP+D3.

For the isolated ADB molecule in solution, the rather large shift of 0.45 eV between absorption and emission maxima could be explained by a reduction of the twist angle between anthracene and phenylene units from 84° to 56° in the excited state equilibrium conformation (see Section 2.1). In the MOF that angle is already decreased to 66° in the ground state, primarily due to the van der Waals interaction between neighboring chromophores (see Section 2.3). Moreover, a further planarization of the ADB linkers incorporated into Zn-ADB SURMOF-2 in the excited state is prevented by steric constraints due to the already tight packing of the ADB chromophores in *c*-direction in the ground state. Indeed, when optimizing the geometry of the (pw-ADB-pw)_4_ tetramer in the S_1_ electronic configuration, yielding ^S1^(pw-ADB-pw)_4_, the changes in tilt angles are only very minor (see Supporting Information SI.7). This applies in particular to the two central pw-ADB-pw units, where the S_1_ state is primarily localized, as can be inferred from the excitation-induced changes in bond lengths and from the transition density shown in Figure 8.

As a consequence, one would expect a smaller shift between the absorption and emission maxima in the MOF. Indeed, when calculating the excited state properties of one of the two central pw-ADB-pw units cut from the optimized S_1_ tetramer, ^S1,cut^(pw-ADB-pw)_1_, one observes an only rather moderately shift of ~0.3 eV (see monomer values in Table 4 and Table 5). A similar shift is actually observed when comparing the energies of the lowest excited states for ^cut^(pw-ADB-pw)_4_ and ^S1^(pw-ADB-pw)_4_. The respective energies amount to 2.86 eV for ground-state conformation (see Table 4) and to 2.49 eV for the excited state conformation (see Table 5), where it should be stressed that both states have the same nature, being dominated by an excitation between equivalent orbitals (see Supplementary Material SI.8). This clearly shows that geometric relaxations in the excited state in Zn-ADB SURMOF-2 would result in a shift between absorption and emission maxima of only half the experimentally observed value of 0.64 eV. 

This suggests that the main features in the emission and absorption spectra are dominated by different electronically excited states: The key difference between them is that the absorption spectrum is influenced by all excited states and dominated by the state(s) with the highest oscillator strength (S_14_ in the case of ^opt^(pw-ADB-pw)_4_). Conversely, what counts for the emission characteristics according to Kasha’s rule are the properties of the lowest excited state, i.e., S_1_. This is in particular the case here, as due to the slip of the anthracene molecules, transitions between this state and the ground state are not optically forbidden in the H-aggregates of Zn-ADB SURMOF-2 (see Section 2.2) [63].

Therefore, to assess the shift between the maxima of the absorption and emission spectra, one has to compare the absorption maximum of ^cut^(pw-ADB-pw)_4_ (which we find at 3.29 eV as discussed in Section 2.4) and the S_1_ energy of ^S1^(pw-ADB-pw)_4_ (of 2.49 eV). This, indeed, yields a red-shift of 0.80 eV, which is consistent with the experimentally observed shift of 0.64 eV. The somewhat larger shift in the calculations occurs not only for Zn-ADB SURMOF-2, but also for ADB in solution (see Table 1). It is mostly due to a minor underestimation of the emission energy, as becomes evident, e.g., from the comparison between the calculated excited state properties and the experimental spectra in the Supplementary Material (SI.9).

In passing we note that even if the lowest excited state was forbidden in absorption, dynamic symmetry breaking due to geometry relaxations in the excited state could relax symmetry-selection rules [63,68]. This is, however, not the case in our TD-DFT simulations, as can be inferred from the reduced oscillator strength of the lowest excited state for the relaxed geometry of ^S1^(pw-ADB-pw)_4_ compared to the ground-state conformation in ^cut^(pw-ADB-pw)_4_. This is potentially a consequence of the delocalization of the exciton over two chromophores.

The above considerations show that the red-shifted emission of Zn-ADB SURMOF-2 is indeed a direct consequence of inter-chromophore interactions, where the subtleties of exciton coupling in slipped chromophores are crucial, while massive, excitation-induced conformational changes, as one would expect in classical excimers, do not play a role.

A final aspect that should be discussed is the possible consequence of the particularly low oscillator strength of 0.06 associated with the S_1_→S_0_ emission transition of ^S1^(pw-ADB-pw)_4_. It implies that the radiative lifetime of Zn-ADB SURMOF-2 should be particularly long, in fact, much longer than the measured overall excited state lifetime of ~4 ns [51] (which would then be determined by the non-radiative lifetime). It also suggests that in case there is some inhomogeneity in the sample including some less well-ordered regions, where the chromophores have a more isolated character, these regions will dominate the emission spectrum immediately after excitations due to the much larger oscillator strengths of isolated chromophores (see Table 1). Only, when the excited state populations in these regions have decayed or when the excitons have migrated to the fully crystalline parts of the samples, the red-shifted emission of the S_1_ state in ^S1^(pw-ADB-pw)_4_ will dominate. Such a red-shift of the emission with time has, indeed, been observed in the experiments on Zn-ADB SURMOF-2 [51].

## 3. Methods

For the investigation of the properties of Zn-ADB SURMOF-2, we performed Density Functional Theory, DFT, calculations on molecules, clusters, and crystalline, 3D periodic structures. All molecule and cluster-based calculations were performed with the ORCA 4.0.1 code [69] employing the PBE0 hybrid functional [65]. In the geometry optimizations of the ground and first excited state, we used the def2-SVP basis set [70] and Grimme‘s D3 dispersion correction [71]. Absorption and emission properties of the relaxed structures were calculated with the linear-response approach within time-dependent DFT (TD-DFT), employing the Tamm-Dancoff approximation (TDA) [72] and using the def2-TZVP basis set [70]. When studying absorption (emission) properties, the lowest twenty (six) excited states were calculated explicitly. To simulate the ADB molecule in solution, solvent effects for ethanol (ε = 24.3) and toluene (ε = 2.4) were included using the SMD continuum solvation model [73].

The periodic structure of the MOF was studied with the program FHI-Aims [74]. We employed a 2 × 2 × 8 k-point grid and *Tight* settings of the numeric atom-centered basis set. A detailed description of the used basis functions is given in the Supplementary Material (SI.1). The atomic ZORA correction was applied for treating relativistic effects [75]. Starting from the experimental XRD-data [51], the cell parameters of the tetragonal unit cell were fixed, while the atom positions within the cell were relaxed until the remaining forces were below 10^−3^ eV/Å. The use of hybrid functionals in conjunction with periodic boundary conditions is prohibitively expensive for systems as large as the MOF studied here. Thus, in the FHI-Aims simulations we employed the Perdew–Burke-Ernzerhof (PBE) functional [64] with the Tkatchenko-Scheffler van der Waals correction [76], where the influence of the functional is addressed in detail in the Supplementary Materials.

Notably, excited state calculations employing DFT and periodic boundary conditions to date are impossible for systems as complex as the ones studied here. Moreover, to the best of our knowledge, no band structure code exist that would let us relax the relevant excited state geometries. Thus, to obtain consistent results, we used the periodic structures only as reference geometries for determining molecular conformations and for extracting starting structures for cluster optimizations.

Such clusters were built from (several) ADB linkers connected to two Zn-paddlewheels (extracted from the FHI-Aims calculations), with each paddlewheel coordinatively saturated with three acetate groups. During the cluster optimizations employing ORCA 4.0.1 and the PBE0 functional, the Zn and O atoms were kept fixed at the positions obtained in the periodic calculation. The geometries of the linkers were fully optimized. When studying clusters representing units of Zn-ADB SURMOF-2, no solvation model was employed, as experiments suggest a solvent free environment inside the MOF [51].

## 4. Conclusions

In this study, we provide a detailed explanation for the excited state properties of anthracene dibenzoic acid (ADB) both as isolated chromophore in solution as well as incorporated as linker into a metal-organic framework (Zn-ADB SURMOF-2). The latter provides a well-controlled arrangement of the chromophores relative to each other in the solid state. The comparison of the two situations (solution and porous solid) is facilitated by the observation that the solvent polarity has virtually no impact on the optical properties of ADB. Within the MOF, H-aggregate type coupling of the ADB entities is identified as the main type of excitonic interaction. Thus, one would expect a distinct blue-shift of the maximum of the absorption spectrum, which is, however, neither observed in the simulations nor in experiments [51]. One of the reasons for that is that in our simulations both the ordering of the excited states as well as the energetic splitting between excited states in the MOF are significantly modified compared to a conventional H-aggregate. This is due to a slip of the centers of neighboring chromophores relative to each other. A second, even more important aspect is that the geometric conformation of the ADB chromophores change considerably inside the MOF due to steric constraints resulting from the rigid network structure. This increases π-conjugation within the ADB linkers and triggers a red-shift of all excited states. The combination of the blue shift due to aggregate formation and the red shift due to conformational changes then leads to the a priori unexpected red-shift of the actual absorption spectrum.

Another striking feature of Zn-ADB SURMOF-2 is the huge shift of 0.64 eV between the maxima of the absorption and emission spectra. This is particularly surprising, as the comparably tight packing of the MOF-linkers in *c*-direction prevents a significant excitation-induced change of the conformation of the individual chromophores. This is in sharp contrast to the situation in solution, where the reduction of the twist between the anthracene and the phenylene units upon excitation provides a major contribution to the red-shift of the emission. There are also no significant changes in the relative arrangement of neighboring chromophores, which could modify the exciton coupling in the excited state. Instead, the highly red-shifted emission of Zn-ADB SURMOF-2 is a consequence of different excited states dominating the absorption and emission spectra. In absorption, primarily strongly allowed states count, which experience a very minor red-shift for the reasons discussed above. Conversely, for the emission, in line with Kasha’s rule, he lowest excited state is the relevant one. Due to the exciton coupling between the strongly interacting linkers in the MOF, this state is strongly red-shifted and as a consequence of the relative slip of the π-planes of neighboring anthracenes, its calculated oscillator strength is small, but non-zero.

These considerations show that it is the subtle interplay of a variety of factors like inter-chromophore couplings, the reordering of states, and packing-induced conformational changes that determine the optical properties of MOFs and that disentangling these factors proofs difficult without performing suitable simulations.

## Figures and Tables

**Table 1:** Experimental and calculated absorption and emission data for ADB in different solvents. For the sake of comparison, also gas-phase absorption data for diphenylanthracene (DPA) and anthracene-dibenzoic acid (ADB) are shown. The calculations have been performed at the PBE0/def2-TZVP//def2-SVP level and the reported values correspond to a purely electronic transition between the ground and the first excited state (S_0_ and S_1_, respectively). The oscillator strengths of the S_0_→S_1_ transitions are given in parentheses. Absorption (emission) data have been calculated for the S_0_ (S_1_) equilibrium geometries. The experimental values for the absorption and emission peaks in ethanol and for the MOF have been extracted from the experimental spectra shown in the Supplementary Materials, which correspond to the spectra shown in [51] (including the respective Supporting Material). Here, in a first approximation, the calculated (vertical) excitation energies are associated with the positions of the maxima of the experimental spectra [54]. The position of the absorption peak in toluene was taken from [55]. For the sake of comparison with calculated data in later sections, the table also contains the experimental peak positions for Zn-ADB SURMOF-2 (i.e., chromophores incorporated into the MOF).

	Energy/eV (Oscillator Strength)	
Absorption	Experiment	Calculation
Anthracene (gas)	-	3.48 (0.085)
DPA (gas)	-	3.36 (0.201)
ADB (gas)	-	3.33 (0.255)
ADB (ethanol ε = 24.3)	3.32	3.35 (0.245)
ADB (toluene ε = 2.4)	3.33	3.35 (0.245)
Zn-ADB SURMOF-2	3.27	
**Emission**		
ADB (gas)	-	2.78 (0.531)
ADB (ethanol)	2.87	2.73 (0.530)
Zn-ADB SURMOF-2	2.63	

**Table 2:** PBE0/def2-TZVP//def2-SVP calculated absorption energies and oscillator strengths (in parentheses) for the most relevant low-energy excitations for a single anthracene molecule, as well as for dimer and tetramer clusters along the three unit cell directions. For the H-aggregates, the S_1_ state and the lowest excited state with appreciable oscillator strength are listed. For the J-aggregate, besides the optically allowed S_1_ state, we also included the next state described by equivalent single-particle excitations (see text). The anthracene units in the model stacks are geometry-optimized at the single-molecule level and then aligned in the same way (center-of-mass distance and tilt angles) as in the MOF optimized using periodic boundary conditions (cf., Figure 3d).

	Energy/eV (Oscillator Strength)		
Monomer	3.48 (0.085)		
	**Unit Cell Direction**		
	**J-aggregate** **(*a* and *b* direction)** **19.81 Å**	**H-aggregate** **(*a* and *b* direction)** **19.81 Å**	**H-aggregate** **(*c*-direction)** **5.82 Å**
Dimer	S_1_: 3.48 (0.172) S_2_: 3.48 (0.000)	S_1_: 3.48 (0.000) S_2_: 3.48 (0.170)	S_1_: 3.16 (0.024) S_4_: 3.55 (0.115)
Tetramer	S_1_: 3.48 (0.333) S_3_: 3.48 (0.014)	S_1_: 3.48 (0.000) S_4_: 3.48 (0.323)	S_1_: 3.06 (0.029) S_14_: 3.60 (0.120)

**Table 3:** PBE0/def2-TZVP//def2-SVP calculated absorption energies and oscillator strengths f (in brackets) of the S_0_-S_1_ transition of coplanar anthracene dimers for different slip distances (see Figure 3d). The orbital contributions are identified following the nomenclature from Figure 4: S = symmetric linear combination, A = antisymmetric linear combination, O = occupied, U = unoccupied.

System	State	Energy/eV (Oscillator Strength)	MO Contributions with Coefficients
anthracene	S_a_	2.75 (0.000)	0.98 OS→UA + 0.16 OA→US
dimer	S_b_	3.11 (0.001)	0.76 OA→UA + 0.65 OS→US
(slip 0.0 Å)	S_c_	3.53 (0.124)	0.72 OS→US − 0.62 OA→UA
	S_d_	3.76 (0.000)	0.97 OA→US − 0.13 OS→UA
anthracene	S_b_	3.16 (0.024)	0.92 OA→UA + 0.36 OS→US
dimer	S_a_	3.22 (0.000)	0.80 OS→UA + 0.59 OA→US
(slip 4.42 Å)	S_d_	3.46 (0.000)	0.78 OA→US − 0.57 OS→UA
	S_c_	3.55 (0.112)	0.91 OS→US − 0.32 OA→UA

**Table 4:** PBE0-D3/def2-TZVP//def2-SVP calculated absorption properties for the monomer ^opt^(pw-ADB-pw)_1_, the dimer ^cut^(pw-ADB-pw)_2_, and the tetramer ^cut^(pw-ADB-pw)_4_ of node-linker-node units in MOF conformation stacked along *c*-direction. The superscripts in the system names denote, how the cluster has been generated. “opt” refers to a full geometry optimization, while “cut” refers to a system, generated by cutting out the central moiety (viz. tet2) of the fully optimized tetramer (see Figure 6c) and replicating it as a dimer or tetramer employing the periodicity of the bulk MOF. The description of the excited states and molecular orbitals in the dimer is analogous to that of the anthracene dimers in Table 3 to highlight the resemblance to the model system. S_a_, S_b_, S_c_, and S_d_ are also the four lowest excited states of ^cut^(pw-ADB-pw)_2_. For the tetramer, all transitions with oscillator strengths greater than 0.15 are shown; there, a nomenclature analogous to the dimer is no longer straightforward. To categorize the molecular orbitals nonetheless, the tetramer was grouped into three pairs of dimers and the symmetry of each pair is given. Again, S denotes a symmetric linear combination and A an antisymmetric one. To better illustrate that, he OAAA and UAAA orbitals for ^cut^(pw-ADB-pw)_4_ are visualized in the Supplementary Material (SI.8).

System	State	Energy/ev (Oscillator Strength)	Mo Contributions with Coefficients
^opt^(pw-ADB-pw)_1_	S_1_	3.35 (0.296)	0.96 H→L
^cut^(pw-ADB-pw)_1_	S_1_	3.18 (0.453)	0.96 H→L
^cut^(pw-ADB-pw)_2_	S_b_	2.93 (0.124)	0.92 OA→UA + 0.33 OS→US
	S_a_	2.99 (0.006)	0.80 OS→UA + 0.58 OA→US
	S_d_	3.14 (0.000)	0.79 OA→US − 0.56 OS→UA
	S_c_	3.26 (0.658)	0.90 OS→US − 0.31 OA→UA
^cut^(pw-ADB-pw)_4_	S_1_	2.86 (0.162)	0.94 OAAA→UAAA
	S_9_	3.22 (0.332)	0.69 OASA→UASA + 0.40 OSAS→USAS
	S_14_	3.32 (0.715)	0.71 OSSS→USSS + 0.47 OSAS→USAS

**Table 5:** PBE0-D3/def2-TZVP//def2-SVP first excited-state properties for the monomer ^S1,cut^(pw-ADB-pw)_1_ and the tetramer ^S1^(pw-ADB-pw)_4_ of node-linker-node units stacked along c-direction. The superscripts in the system definition denote, how the cluster has been generated. “S1” denotes a full geometry optimization in the first excited state, while “cut” refers to system generated by cutting out the central moiety, tet2, of the S_1_ fully optimized tetramer analogous to Figure 6c. The description of the molecular orbital contributions of the S_1_ optimized tetramer is analogous to that in Table 4.

System	State	Energy/ev Oscillator Strength	Mo Contributions with Coefficients
^S1,cut^(pw-ADB-pw)_1_	S_1_	2.90 (0.557)	0.96 H→L
^S1^(pw-ADB-pw)_4_	S_1_	2.49 (0.060)	0.93 OAAA→UAAA

## Supplementary Materials

The following are available online. SI.1 FHI-Aims basis set; SI.2 Ground state geometry of ^opt^(pw-ADB-pw)_1_; SI.3 Ground state geometry of Zn-SURMOF2 and influence of van der Waals interaction; SI.4 Ground state geometry of (pw-ADB-pw)_4_ compared to the periodic calculations – impact of the choice of the functional; SI.5 Dependence of the absorption of ADB on the anthracene-phenylene angle; SI.6 Calculated absorption spectra of various MOF models; SI.7 Structural properties of ^S1^(pw-ADB-pw)_4_; SI.8 Orbitals most relevant for the lowest-lying excited states in ^cut^(pw-ADB-pw)_4_ and ^S1^(pw-ADB-pw)_4_; SI.9 Comparison between calculated excitation energies and oscillator strengths and experimental spectra in solution and for Zn-ADB SURMOF-2; geometries of all systems used in the simulations.
