# Supplementary material for: Exciton Coupling and Conformational Changes Impacting the Excited State Properties of Metal Organic Frameworks
Source: Molecules. 2020 Sep 15;25(18):4230. doi: 10.3390/molecules25184230 (PMC7570727; doi:10.3390/molecules25184230)
Supplement: Supplementary file 1 [file molecules-25-04230-s001.zip › molecules-930683-supplementary-final.docx]

**Supplementary Material for**

**Exciton Coupling and Conformational Changes Impacting the Excited State Properties of Metal Organic Frameworks**

Andreas Windischbacher ^1,2,3^, Luca Steiner ^1^, Ritesh Haldar ^4^, Christof Wöll ^4^, Egbert Zojer ^2*^, Anne-Marie Kelterer ^1*^

^1^ Institute of Physical and Theoretical Chemistry, Graz University of Technology, Stremayrgasse 9, 8010 Graz, Austria

^2^ Institute of Solid State Physics, Graz University of Technology, Petersgasse 16, 8010 Graz, Austria

^3^ Institute of Physics, University of Graz, Universitätsplatz 5, 8010 Graz, Austria

^4^ Institute of Functional Interfaces (IFG), Karlsruhe Institute of Technology (KIT) ,Hermann-von-Helmholtz Platz-1, 76344 Eggenstein-Leopoldshafen, Germany

Corresponding authors: Anne-Marie Kelterer, [kelterer@tugraz.at](mailto:kelterer@tugraz.at), Egbert Zojer, egbert.zojer@tugraz.at

**SI.1 FHI-Aims basis set**

Table S1 lists the basis functions that build up the *tight* basis set of FHI-Aims used in the calculations of the Zn-MOF. The abbreviations read as follows: X(n,l,z), where X can either be H for hydrogen-like type function or I for ionic-like type function, n is the main quantum number, l is the angular momentum quantum number and z is an effective nuclear charge.

Table S1. Basis functions that build up the tight basis set of FHI-Aims used in the periodic calculations.

|  | H | C | O | Cu | Zn |
| --- | --- | --- | --- | --- | --- |
| Minimal | 1s | [He] 2s2p | [He] 2s2p | [Ar] 3d4s | [Ar] 3d4s |
| Tier 1 | H(2s,2.1)  H(2p,3.5) | H(2p,1.7)  H(3d,6)  H(2s,4.9) | H(2p,1.8)  H(3d,7.6)  H(3s,6.4) | I(4p,auto)  H(4f,7.4)  H(3s,2.6)  H(3d,5)  H(5g,10.4) | H(2p,1.7)  H(3s,2.9)  H(4p,5.4)  H(4f,7.8)  H(3d,4.5) |
| Tier 2 | H(1s,0.85)  H(2p,3.7)  H(2s,1.2)  H(3d,7) | H(4f,9.8)  H(3p,5.2)  H(3s,4.3)  H(5g,14.4)  H(3d,6.2) | H(4f,11.6)  H(3p,6.2)  H(3d,5.6)  H(5g,17.6)  H(1s,0.75) | - | - |

**SI.2 Ground state geometry of ^opt^(pw-ADB-pw)_1_**

Table S2 lists selected geometric parameters, referred to in all geometry comparisons: Ant/Ph corresponds to the angle between the anthracene and the phenyl rings. Zn/Ant and Zn/Ph correspond to the angles between the plane of the carboxylic groups and the anthracene and phenyl rings, respectively. The bonds are numbered according to Figure S1.

After optimization of the ground state of an ADB molecule connected to two Zn-paddlewheels, ^opt^(pw-ADB-pw)_1_, the conformation of the ADB moiety is still in good agreement with that of an isolated molecule. This shows that the Zn-nodes have little influence on the conformation of the linker and, additionally, it points out the importance of periodic calculations. Only when considering the 3D structure of the MOF, changes in the chromophore conformation become apparent. The small differences in bond lengths arise as the positions of the Zn-nodes were kept fix to the positions they adopt in the periodic structure calculation of the MOF, thus slightly elongating the single bonds of the molecule.


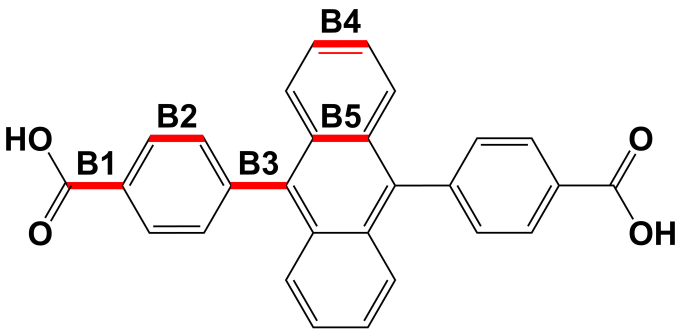


Figure S1. Selected bonds of ADB, which are used for comparison of geometric parameters

Table S2. Dihedral angles and bond lengths of the ground state optimized ^opt^(pw-ADB-pw)_1_ unit compared to the ADB molecule (both PBE0/def2-svp+D3).

| **S_0_** | **ADB molecule** | **^opt^(pw-ADB-pw)_1_** |
| --- | --- | --- |
| Ant/Ph [°] | 82.0 | 80.7 |
| Zn/Ant [°] | 82.2* | 82.0 |
| Zn/Ph [°] | 0.2* | 1.3 |
| B1 [Å] | 1.486 | 1.511 |
| B2 [Å] | 1.390 | 1.396 |
| B3 [Å] | 1.488 | 1.508 |
| B4 [Å] | 1.421 | 1.420 |
| B5 [Å] | 1.441 | 1.450 |

*as Zn atoms are missing, the angles are measured relative to the O-plane

**SI.3 Ground state geometry of Zn-SURMOF2 and influence of van der Waals interaction**

Compared to a diluted solution, the ADB molecules are rather closely packed in the periodic framework, especially in c-direction. To show the influence of the periodicity on the structure, the geometry of the periodic calculation is compared to the molecular simulation. Table S3 lists the dihedral angles between the functional moieties of ADB and selected bond lengths according to Figure S1. Furthermore, we also did tests switching off van der Waals corrections in the periodic calculation to highlight the importance of dispersion interactions for the linker conformation. Note, that the periodic calculations employed the PBE functional, while molecular calculations were obtained on a PBE0 level of theory.

As we have taken the experimental unit cell of Zn-SURFMOF2, the single bonds of the ADB molecule are slightly stretched. Upon incorporation into the periodic framework, the phenyl rings tilt out of the COO-plane due to the steric repulsion of the neighbouring linker units. As can be seen from the two periodic calculations, the rotation of the anthracene is mainly due to attractive dispersion forces. Without van der Waals forces, the Ant/Ph angle of the linker is similar to the corresponding angle in the molecule.


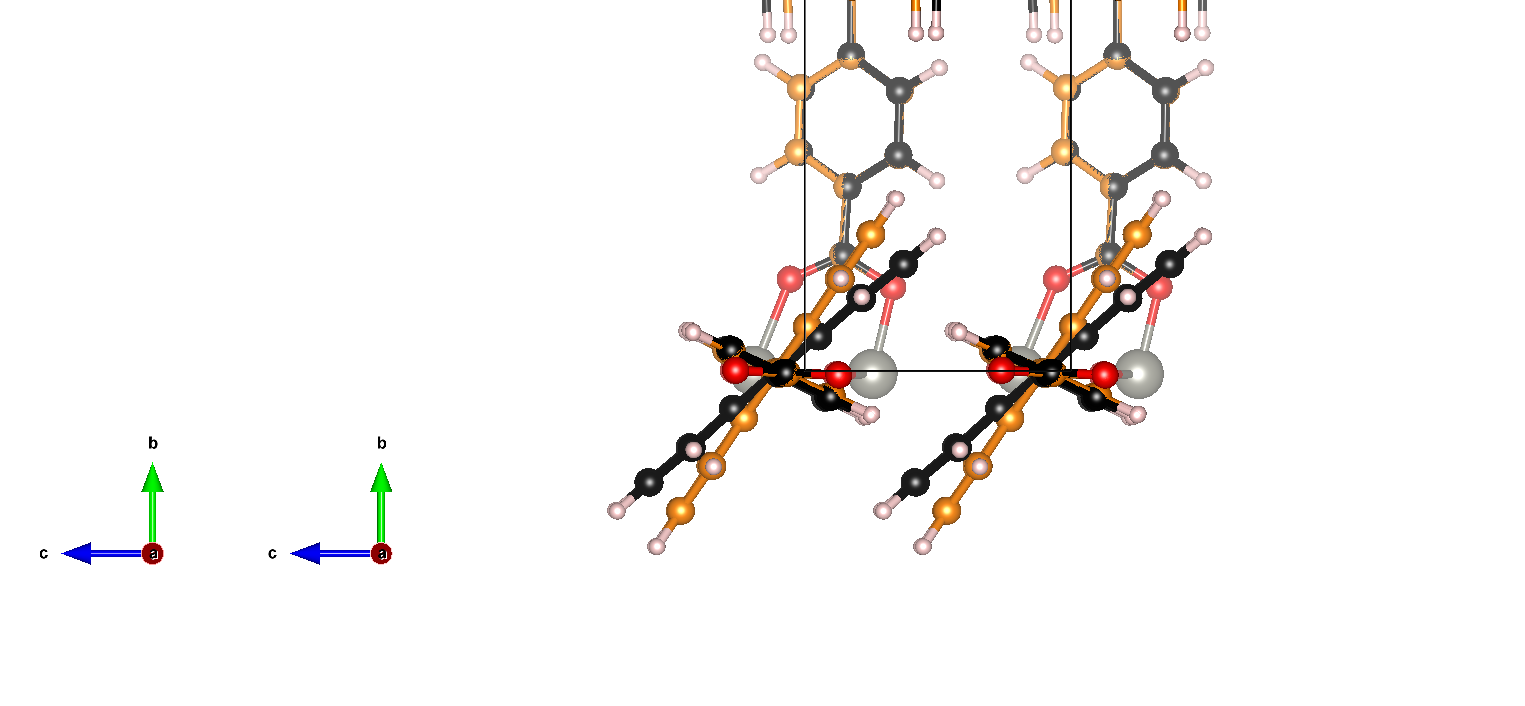


Figure S2. Detail of the unit cell of Zn-ADB-SURMOF2 with (black) and without (orange) van der Waals correction (Tkatchenko-Scheffler).

Table S3. Comparison of selected geometric parameters of ADB molecules (ORCA, PBE0/def2-SVP+D3) and the periodic bulk structure in experimental unit cell (FHI-AIMS, PBE/tight+TS).

| **S_0_** | **periodic** | | **ADB molecule** | |
| --- | --- | --- | --- | --- |
|  | ***vdW*** | ***without vdW*** | |  |
| Ant/Ph [°] | 66.3 | 80.1 | | 82.0 |
| Zn/Ant [°] | 42.3 | 57.0 | | 82.2* |
| Zn/Ph [°] | 24.0 | 23.1 | | 0.2* |
| B1 [Å] | 1.509 | 1.512 | | 1.486 |
| B2 [Å] | 1.400 | 1.401 | | 1.390 |
| B3 [Å] | 1.507 | 1.510 | | 1.488 |
| B4 [Å] | 1.417 | 1.419 | | 1.421 |
| B5 [Å] | 1.455 | 1.456 | | 1.441 |

*as Zn atoms are missing, the angles are measured relative to the O-plane

**SI.4 Ground state geometry of (pw-ADB-pw)_4_ compared to the periodic calculations – impact of the choice of the functional**

Reducing the periodic environment to a cluster of finite size can be justified by a good resemblance of the linker-node conformation in the optimized cluster compared to the periodic framework. The dihedral angles and selected bond lengths of the linkers calculated with periodic boundary conditions are compared to the four units of a tetramer, ^opt^(pw-ADB-pw)_4_, along the parallel stacking direction (c-direction of the unit cell) in Table S4. Note, that the periodic calculations employed the PBE functional, while molecular calculations were obtained at a PBE0 level of theory. Differences in bond lengths between the periodic PBE and cluster PBE0 calculations are very small. This is to a large extent due to fixing the positions of the Zn-nodes to the results of the periodic simulations, where the unit-cell dimensions are fixed to the experimental data.. Both central units of the cluster (tet2 and tet3 in Figure S3) adopt a conformation consistent with the periodic simulations. In comparison, the outer units (tet1 and tet4) undergo a twist of the ADB linker towards smaller Zn/Ph angles like for the fully optimized unit, ^opt^(pw-ADB-pw)_1_ (cf. Table S2). We attribute that to edge effects due to the missing neighbors.

**
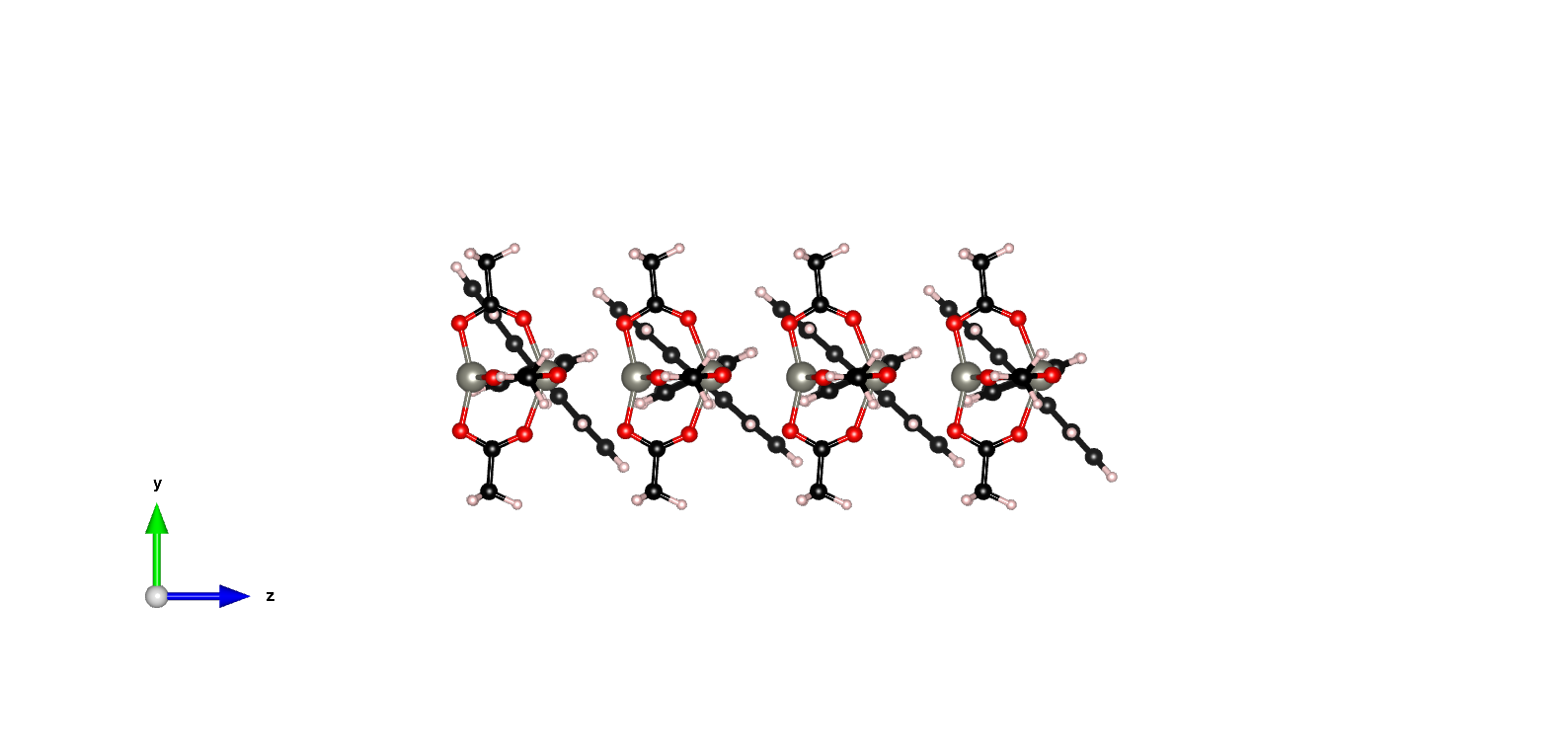
**

**tet1 tet2 tet3 tet4**

Figure S3. Labelling of the monomer units in a tetramer calculation.

Table S4. Comparison of selected geometry parameters of ^opt^(pw-ADB-pw)_4_ (ORCA, PBE0/def2-svp+D3) with periodic structure (FHI-AIMS, PBE/tight+TS), labelling of the units according to Figure S3.

| **S_0_** | **periodic** | ***^opt^(pw-ADB-pw)_4_*** | | | |
| --- | --- | --- | --- | --- | --- |
|  |  | **tet 1** | **tet 2** | **tet 3** | **tet 4** |
| Ant/Ph [°] | 66.3 | 66.5 | 64.9 | 64.1 | 65.7 |
| Zn/Ant [°] | 42.3 | 51.4 | 41.9 | 42.2 | 46.8 |
| Zn/Ph [°] | 24.0 | 15.1 | 23.0 | 21.9 | 19.0 |
| B1 [Å] | 1.509 | 1.512 | 1.512 | 1.511 | 1.512 |
| B2 [Å] | 1.400 | 1.397 | 1.396 | 1.395 | 1.395 |
| B3 [Å] | 1.507 | 1.512 | 1.510 | 1.510 | 1.510 |
| B4 [Å] | 1.417 | 1.419 | 1.418 | 1.418 | 1.418 |
| B5 [Å] | 1.455 | 1.453 | 1.452 | 1.452 | 1.452 |

**SI.5 Dependence of the absorption of ADB on the anthracene-phenylene angle**

To confirm the correlation between the red-shift and the decreased twist between anthracene and phenylenes, we artificially changed the twist angle in ^cut^(pw-ADB-pw)_1_ back to the value obtained in the isolated system (from 66° to 84°). This yields optical properties very similar to the isolated ADB molecule (see Table S5). This shows that the reduced twist between the anthracene and the phenylenes in ^cut^(pw-ADB-pw)_1_ is primarily responsible for the red-shift of the absorption spectrum in the MOF-conformation of ADB. The same behavior is also observed for the dimer.

Table S5: PBE0-D3/def2-TZVP//def2-SVP absorption properties for the artificially rotated monomer, ^84^(pw-ADB-pw)_1_, and dimer ^84^(pw-ADB-pw)_2_ compared to the optimized monomer ^opt^(pw-ADB-pw)_1_.

| system | state | energy / eV (oscillator strength) | MO contributions with coefficients |
| --- | --- | --- | --- |
| ^opt^(pw-ADB-pw)_1_ | S_1_ | 3.35 (0.296) | 0.96 H→L |
| ^84^(pw-ADB-pw)_1_ | S_1_ | 3.34 (0.299) | 0.96 H→L |
| ^84^(pw-ADB-pw)_2_ | S_b_ | 3.14 (0.000) | 0.92 OA→UA + 0.33 OS→US |
|  | S_a_ | 3.16 (0.000) | 0.80 OS→UA + 0.58 OA→US |
|  | S_d_ | 3.31 (0.000) | 0.79 OA→US – 0.56 OS→UA |
|  | S_c_ | 3.36 (0.532) | 0.90 OS→US – 0.31 OA→UA |

To further visualize the change of the transition density accompanied with the reduced twist angle, we integrate the transition density over the two short axes of the linker-node unit. Figure S4 shows the integrated densities of two linker-node units with different twist angles, ^opt^(pw-ADB-pw)_1_ (ant/ph = 84°) and ^cut^(pw-ADB-pw)_1_ (ant/ph = 66°). By decreasing the twist angle between anthracene and phenylenes, the transition density on the anthracene moiety decreases while the density on the phenylenes increases.


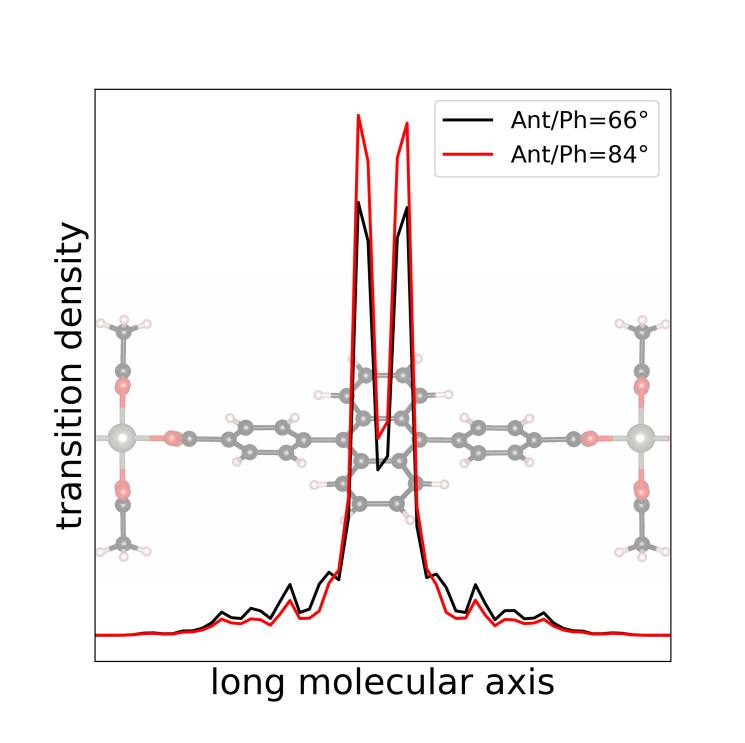


Figure S4. Plane-integrated transition densities for a single pw-ADB-pw unit with Ant/Ph angles of 66° (^cut^(pw-ADB-pw)_1_) and 84°( ^opt^(pw-ADB-pw)_1_). The integration is performed over a plane parallel to the long axis of the anthracene molecule and perpendicular to its short axis. The linker-node unit is shown in the background to illustrate, which parts of the linker the specific transition densities are associated with.

**SI.6 Calculated absorption spectra of various MOF models**

Comparing calculated excitation energies to experimental absorption maxima can be not straightforward, when excitations to several states contribute to a single absorption peak. Therefore, we show the simulated absorption spectra of the optimized node-linker unit ^opt^(pw-ADB-pw)_1_ as well as of various clusters in MOF conformation in Figure S5. These spectra have been obtained from a superposition of Gaussian peaks with full widths at half maximum (FWHM) of 0.3 eV centered at the energies of the respective excited states and scaled by their oscillator strengths (cf. Table S6). One observes a distinct red-shift between ^opt^(pw-ADB-pw)_1_ and ^cut^(pw-ADB-pw)_1_ due to the planarization of the ADB moiety. When considering the periodic environment via neighboring units in ^cut^(pw-ADB-pw)_2_ and ^cut^(pw-ADB-pw)_4_, the absorption maximum shifts to larger energies due to H-aggregation.


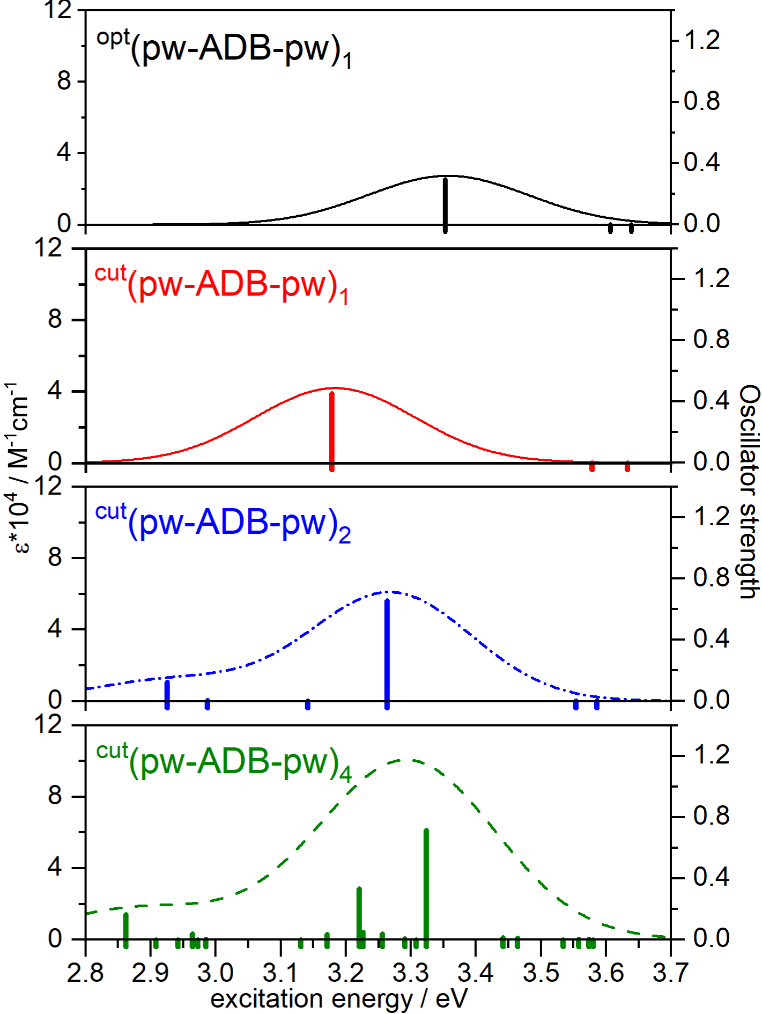


Figure S5. PBE0/def2-tzvp/def2-svp simulated absorption spectra of various model chromophore configurations obtained via convolution with Gaussian peaks (FWHM=0.3 eV); ε denotes the attenuation coefficient. Additionally, the positions of the electronically excited states are shown via the vertical bars, whose (positive) lengths describe the oscillator strengths of the corresponding transitions. The extent of the bars to negative values serves to also show the energetic positions of states with vanishing oscillator strength.

Table S6. Peak maxima of the PBE0/def2-tzvp/def2-svp simulated absorption spectra of various model chromophore configurations obtained via convolution with Gaussian peaks (FWHM=0.3 eV). The corresponding attenuation coefficient (ε) is given in brackets.

| system | peak maximum / eV (ε*10^4^ / M^-1^cm^-1^) |
| --- | --- |
| ^opt^(pw-ADB-pw)_1_ | 3.36 (2.7) |
| ^cut^(pw-ADB-pw)_1_ | 3.18 (4.2) |
| ^cut^(pw-ADB-pw)_2_ | 2.92 (1.3)  3.27 (6.1) |
| ^cut^(pw-ADB-pw)_4_ | 2.88 (1.9)  3.29 (10.1) |

**SI.7 Structural properties of ^S1^(pw-ADB-pw)_4_**

Table S7 lists selected geometric parameters of the ADB molecule and the tetramer (pw-ADB-pw)_4_, both optimized for the first excited state (S_1_) conformation. Optimization of the first excited state of (pw-ADB-pw)_4_ leads to rather small changes in the dihedral angles of the four linker units compared to the ground state configuration ^opt^(pw-ADB-pw)_4_ (compare Table S4 and Table S7). These changes are much less pronounced than for the isolated molecule, as in the MOF the twist angle between the anthracene and phenylene units in the ADB linker has already been reduced due to dispersive interactions and the rather dense packing in the periodic framework. Nevertheless overall, the two central units show a slightly stronger planarization than the edge units with an Ant/Ph angle close to the one of the S_1_ state of the optimized molecule. This suggests a localization of the excited state on the central two chromophore units consistent with the conclusion from the changes in bond lengths between the ground and first excited discussed the main manuscript (also compare Table S4 and Table S7).

Table S7. Dihedral angles of the first excited state optimized ^S1^(pw-ADB-pw)_4_ compared to the ADB molecule (both PBE0/def2-svp+D3), labelling according to Figure S3.

| **S_1_** | **ADB molecule** | ***^S1^(pw-ADB-pw)_4_*** | | | |
| --- | --- | --- | --- | --- | --- |
|  |  | **tet 1** | **tet 2** | **tet 3** | **tet 4** |
| Ant/Ph [°] | 58.6 | 66.0 | 59.2 | 62.0 | 66.6 |
| Zn/Ant [°] | 58.3* | 52.1° | 41.8 | 37.7 | 50.5 |
| Zn/Ph [°] | 0.8* | 17.5° | 20.2 | 26.8 | 18.2 |
| B1 [Å] | 1.476 | 1.511 | 1.499 | 1.518 | 1.514 |
| B2 [Å] | 1.386 | 1.396 | 1.393 | 1.396 | 1.394 |
| B3 [Å] | 1.467 | 1.512 | 1.498 | 1.505 | 1.510 |
| B4 [Å] | 1.395 | 1.418 | 1.400 | 1.400 | 1.419 |
| B5 [Å] | 1.440 | 1.452 | 1.451 | 1.449 | 1.453 |

*as Zn atoms are missing, the angles are measured relative to the O-plane

**SI.8 Orbitals most relevant for the lowest-lying excited states in ^cut^(pw-ADB-pw)_4_ and ^S1^(pw-ADB-pw)_4_**

To show that the first excited states in ^cut^(pw-ADB-pw)_4_ and ^S1^(pw-ADB-pw)_4_ have the same nature, isodensity plots of the orbitals dominating their description are shown in Figure S6.


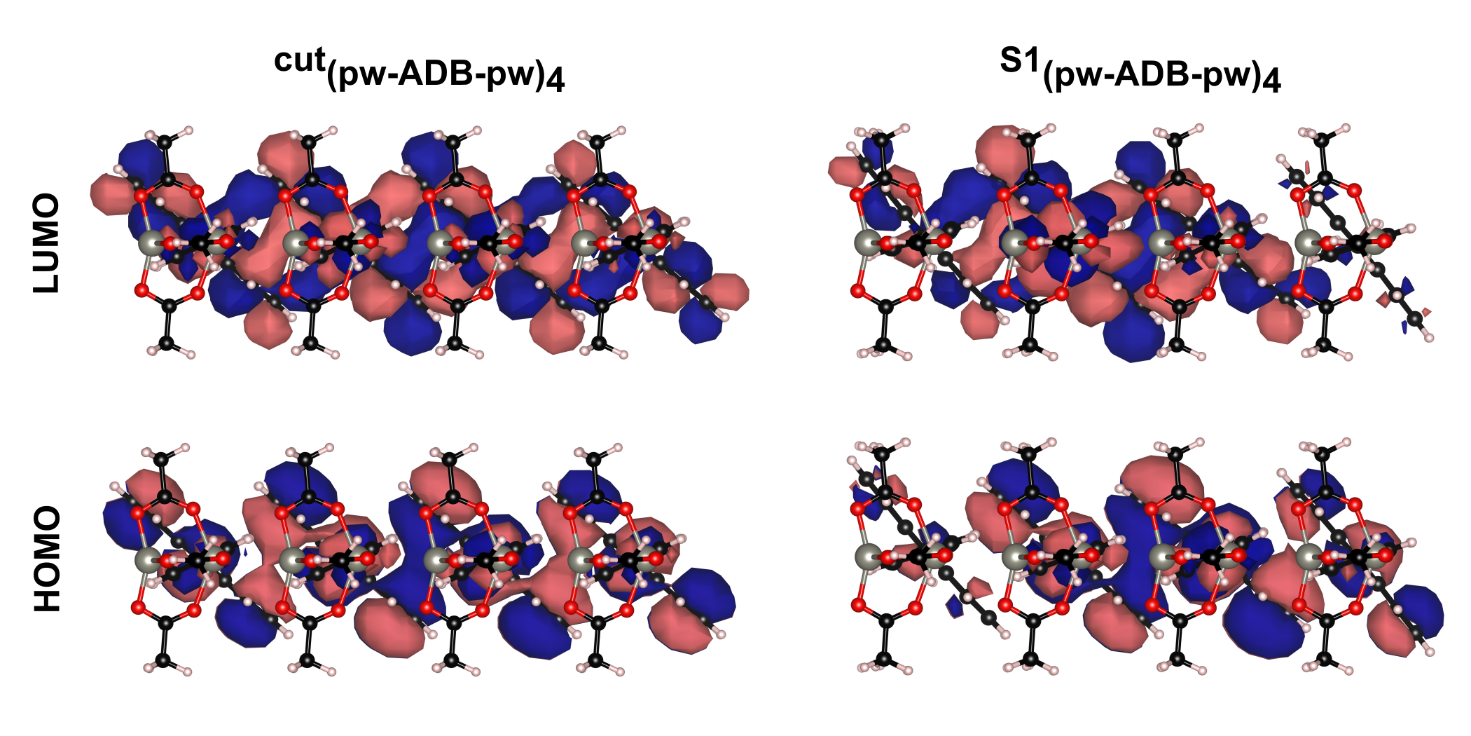


Figure S6. Isodensity plots (isovalue 0.005) of the frontier orbitals of ^cut^(pw-ADB-pw)_4_ and ^S1^(pw-ADB-pw)_4_ illustrating their equivalent nature. These orbitals dominate the description of the S1 state in both systems.

**SI.9 Comparison between calculated excitation energies and oscillator strengths and experimental spectra in solution and for Zn-ADB SURMOF-2**

Figure S7 shows a comparison between the calculated and measured optical properties of ADB in solution and when incorporated into Zn-ADB SURMOF-2. As the calculations only consider purely electronic excitations and, thus, cannot reproduce the vibronic progressions seen in the experiments, they have to be considered as an approximation to the maxima of the experimental spectra.[1]


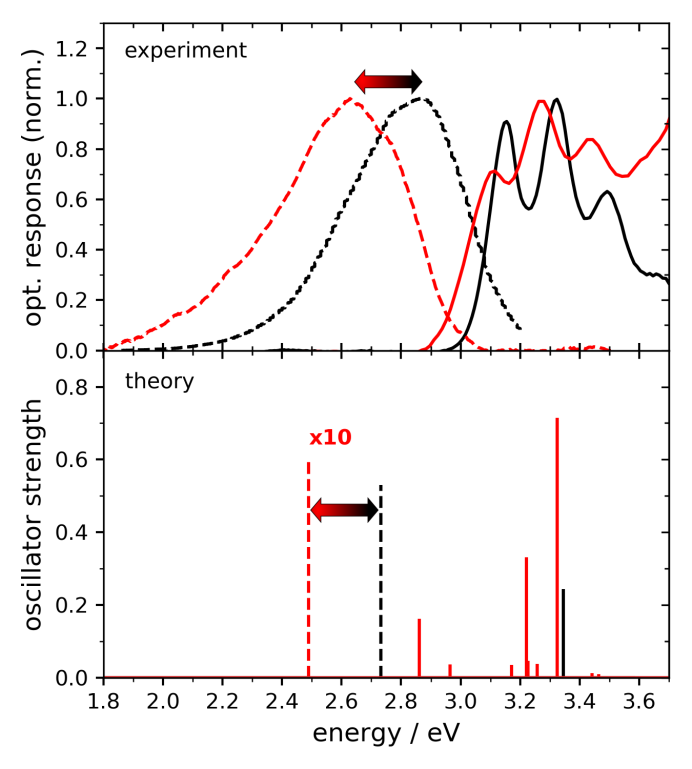

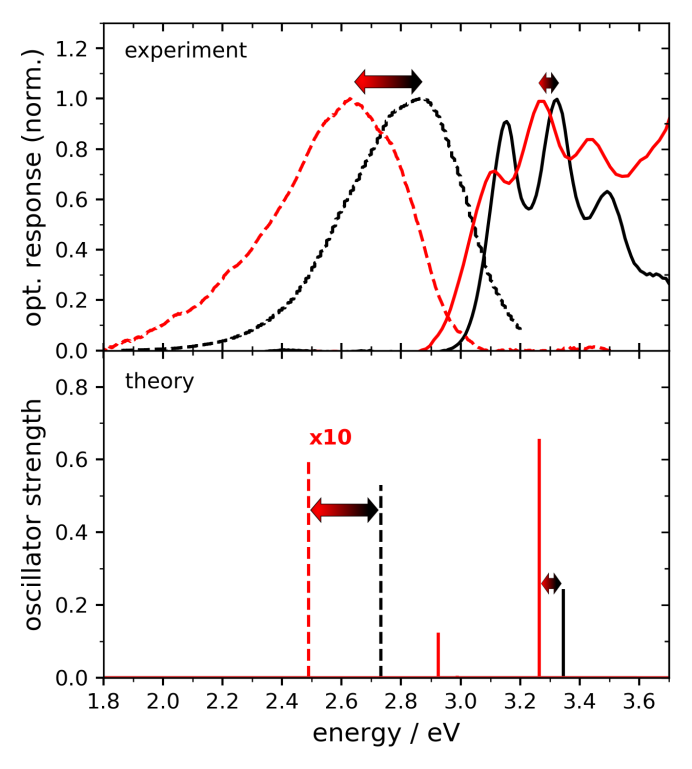


Figure S7. Top panel shows the measured absorption (solid line) and emission spectra (dashed line) for ADB in ethanol solution (black) and incorporated into Zn-ADB SURMOF-2 (red). Bottom panel shows the PBE0/def2-tzvp/def2-svp calculated excitation energies and oscillator strengths. (same color code). For the absorption transitions the results for the dimer (right panel) and for the tetramer (left panel) are shown. For the emission only the properties of the S1 state are shown, as according to Kasha’s rule this state dominates the emission process. The colored arrows in each panel have exactly the same length to allow a quantitative comparison of calculated and measured shifts. Such a comparison is not possible for the ^cut^(pw-ADB-pw)_4_ simulation as there, one encounters two close-lying excited states with similar oscillator strengths. The data correspond to those shown in the Supporting Information of [2], where also details of the experimental procedure for obtaining the spectra can.

The comparison in Figure S7 shows that the overall agreement between theory and experiments is excellent, as suggested already by the numerical values discussed in the main manuscript. The energies of the relevant excited states are close to the maxima of the spectra, with a minor underestimation of the energies of the emission transition in the simulations. This results in a good correspondence between calculated and measured shifts between emission and absorption maxima. Especially the magnitude of the peak shifts between solution and Zn-ADB SURMOF-2 are very well reproduced in the simulations (see colored arrows in Figure S7). The only more serious deviation between theory and experiment is that for the absorption spectra there is no direct experimental evidence for the (rather weakly) allowed transition to the S_1_ state. A comparison between the results for ^cut^(pw-ADB-pw)_4_ and ^cut^(pw-ADB-pw)_2_ shows that the exact position of that state as well as its oscillator strength is distinctly influenced by the considered model configuration.

***References***

[1] J. Gierschner, J. Cornil, and H. J. Egelhaaf, “Optical bandgaps of π-conjugated organic materials at the polymer limit: Experiment and theory,” *Adv. Mater.*, vol. 19, no. 2, pp. 173–191, 2007, doi: 10.1002/adma.200600277.

[2] R. Haldar, M. Jakoby, A. Mazel, Q. Zhang, A. Welle, T. Mohamed, P. Krolla, W. Wenzel, S. Diring, F. Odobel, B. S. Richards, I. A. Howard, and C. Wöll, “Anisotropic energy transfer in crystalline chromophore assemblies,” *Nat. Commun.*, vol. 9, no. 1, pp. 1–8, 2018, doi: 10.1038/s41467-018-06829-3.
